# Preliminary study of relationships between hypnotic susceptibility and personality disorder functioning styles in healthy volunteers and personality disorder patients

**DOI:** 10.1186/1471-244X-11-121

**Published:** 2011-07-30

**Authors:** Fenghua Wang, Wanzhen Chen, Jingyi Huang, Peiwei Xu, Wei He, Hao Chai, Junpeng Zhu, Wenjun Yu, Li Chen, Wei Wang

**Affiliations:** 1 Department of Medical Psychology, School of Public Health, Harbin Medical University, Harbin, China; 2 Department of Clinical Psychology and Psychiatry, Zhejiang University School of Medicine, Hangzhou, China; 3 Department of Preventive Medicine, Jiaxing University School of Medicine, Jiaxing, China; 4 Key Laboratory of Medical Neurobiology of Chinese Ministry of Health, Hangzhou, China

**Keywords:** Hypnotic susceptibility, Personality disorder functioning style, Posthypnotic amnesia, Taste hallucination

## Abstract

**Background:**

Hypnotic susceptibility is one of the stable characteristics of individuals, but not closely related to the personality traits such as those measured by the five-factor model in the general population. Whether it is related to the personality disorder functioning styles remains unanswered.

**Methods:**

In 77 patients with personality disorders and 154 healthy volunteers, we administered the Stanford Hypnotic Susceptibility Scale: Form C (SHSSC) and the Parker Personality Measure (PERM) tests.

**Results:**

Patients with personality disorders showed higher passing rates on SHSSC Dream and Posthypnotic Amnesia items. No significant correlation was found in healthy volunteers. In the patients however, SHSSC Taste hallucination (β = 0.26) and Anosmia to Ammonia (β = -0.23) were significantly correlated with the PERM Borderline style; SHSSC Posthypnotic Amnesia was correlated with the PERM Schizoid style (β = 0.25) but negatively the PERM Narcissistic style (β = -0.23).

**Conclusions:**

Our results provide limited evidence that could help to understand the abnormal cognitions in personality disorders, such as their hallucination and memory distortions.

## Background

Hypnotic susceptibility is an inherent capacity or ability of an individual to experience hypnosis [1]. Although being a stable characteristic [2], it hardly correlates with normal personality traits [3,4]. In clinics however, higher hypnotic susceptibility has been reported in patients suffering from posttraumatic stress disorder (PTSD) [5], acute stress disorder [6], or dissociative identity disorder (DID, also known as multiple personality disorder) [7,8], while lower in schizophrenia patients [7,9]. One possible reason for these discrepancies might be due to the different instruments used to assess the hypnotic susceptibility [9]. Another reason might be related to the personality traits, or their disordered forms (personality disorders) of the participants included in these studies. Indeed, personality disorders were often comorbid with Axis I disorders such as schizophrenia, PTSD, or DID [10,11]. Nevertheless, up to now, no study has been designed to investigate the relationships between hypnotic susceptibility and personality disorders.

There are many scales assessing hypnotic susceptibility. However, most of these, although heterogeneous, cover at least two out of the three main factors of hypnosis: (1) responding to calls for motor performance (direct suggestion, such as lowering your hand), (2) performing certain acts (loss of arbitrary motor control, such as inability to lift your hand), and (3) responding to suggestion for changes in participants' perception, memory, and cognition. One such assessment is the Stanford Hypnotic Susceptibility Scale: Form C (SHSSC) [12], which included all three factors, and has been extensively used in searching for relationships between hypnotic susceptibility and normal personality traits in general populations and in clinics [13,14].

On the other hand, although there are many instruments developed to measure personality disorders in clinics in a categorical way, the Parker Personality Measure (PERM) [15] has proven to be reliable to measure 11 functioning styles of personality disorder. These functioning styles loaded on a dimensional layout, i.e., the disordered personality traits in a predictable way [16].

The present study was designed to determine whether personality disorder patients have different hypnotic susceptibilities when compared to healthy volunteers, and what the relationships are between hypnotic susceptibility (as measured by SHSSC) and personality disorder functioning styles (as measured by PERM). We hypothesized that: (1) personality disorder patients would have higher hypnotic susceptibilities than normal controls; and (2) personality disorder functioning styles would be correlated with hypnotic susceptibilities.

## Methods

### Participants

Initially, we invited 162 university students and 82 personality disordered patients to participate in this study. After we explained the research goals, general procedures, and potential impact, eight healthy university volunteers and five personality disorder patients withdrew from the experiment, stating that they were reluctant to experience hypnosis. In total, 154 healthy volunteers (102 men; aged 20.74 years ± 1.43 S.D., ranged 18 ~ 25 years), and 77 personality disorder patients (51 men; aged 20.58 ± 1.24, ranged 18 ~ 23) were included without receiving any incentive for their participation. A semi-structured interview was performed with each healthy participant to ensure that they were not suffering from any psychiatric or neurological problem. All patients with personality disorders were categorically diagnosed by an experienced psychiatrist using DSM-IV-TR criteria [17] and later with SCID-II for confirmation. Moreover, Computer Tomography or Magnetic Resonance Imaging scans conducted on all patients had displayed normal skulls, midlines, parenchyma, including cerebella and brain stems, and no organic brain lesions were found. All patients were comorbid with Axis I disorders, such as depression, anxiety or sleep disorder, but they were free from DID, drug/alcohol abuse, and schizophrenia. All participants were requested to refrain from consuming any drugs or alcohol for at least 72 hours prior to the test. No significant difference was found between the two groups regarding either age (t = 0.14, 95% CI: -0.35 ~ 0.41, p > 0.05) or gender (χ^2 ^= 0.00, OR = 1.00, 95% CI: -0.56 ~ 1.78, p = 1.00). The study was approved by the Ethic Committee of Zhejiang University School of Medicine, and all participants gave their written informed consent to participate.

### Measures

The participants were asked to undergo the Stanford Hypnotic Susceptibility Scale: Form C (SHSSC) test, and to complete the Parker Personality Measure (PERM) in a quiet room.

#### SHSSC

The Chinese version of the SHSSC was translated from (and back-translated to) the original English version [12] by a Professor and two PhD candidates majoring in Clinical Psychology and Psychiatry. The SHSSC was administered to the participants person by person, beginning with a hypnotic introduction that instructed them to relax and to close their eyes.

Participants' behaviors were scored according to the criteria below:

One point was given to: (1) **Hand lowering **(right hand) if hand had lowered at least 15 cm by end of 10 seconds; (2) **Moving hands apart **if hands were 15 cm or more apart at end of 10 seconds; (3) **Mosquito hallucination **for any grimacing, movement, or acknowledgment of effect (feeling of mosquito); (4) **Taste hallucination **if both sweet and sour tastes were experienced and either one strong or one with movements; (5) **Arm rigidity **if there was less than 5 cm of arm bending in 10 seconds; (6) **Dream **if participant dreamed well (i.e., has an experience comparable to a dream); (7) **Age regression **if there were clear changes in handwriting between the present and one of the regressed ages; (8) **Arm immobilization **(left arm) if arm raised less than 2.5 cm in 10 seconds; (9) **Anosmia to ammonia **if odor of ammonia denied and overt signs absent; (10) **Hallucinated voice **if participant answered realistically at least once (the question voice did not exist actually); (11) **Negative visual hallucination **if hallucination was present, whether or not sustained (see two boxes, actually three); (12) **Posthypnotic amnesia **if participant recalled three or fewer items before "Now you can remember everything".

If a participant got one point on an item, we referred to this as "he/she has passed the item", otherwise as "he/she has failed to pass the item". We defined "passing rate" of each item as the percentage of the participants who have passed the item.

#### PERM

PERM has 92 items drawn from several descriptor pools for personality disorders, such as the International Classification of Diseases and the DSM systems, and the Schedule for Normal and Abnormal Personality, measuring 11 functioning styles of personality disorder: the Paranoid, Schizoid, Schizotypal, Antisocial, Borderline, Histrionic, Narcissistic, Avoidant, Dependent, Obsessive-Compulsive and Passive-Aggressive styles. Each PERM item consists a 5-point Likert scale (1 - very unlike me, 2 - moderate unlike me, 3 - somewhat unlike and like me, 4 - moderate like me, 5 - very like me). The Chinese version of PERM has previously been shown to be reliable in a Chinese sample [16].

### Statistic analyses

SPSS 16.0 was used for statistical analyses. Repeated analyses of variance (ANOVA) plus post-hoc analysis by Dunnett's multiple new range test were applied to PERM scales in two groups. The passing rates of the 12 SHSSC items were analyzed by Chi-Square test. We administered multiple linear regression analyses (step-wise method) in both groups to further explore the relationships between SHSSC and PERM scales, i.e., the prediction of personality functioning styles by SHSSC items.

## Results

The internal reliability for the SHSSC in the current study was 0.72, and those for the 11 PERM scales ranged from 0.55 to 0.80, which were similar to those in Wang et al. [16].

The PERM scale scores were statistically significantly different between the two groups (see Table 1). Post-hoc analyses also showed that patients scored significantly higher than the healthy volunteers on all 11 PERM scales (see Table 2). When comparing the passing rates of SHSSC items, patients passed the Dream (χ^2 ^= 3.97, OR = 1.75, 95% CI: 1.01 ~ 3.06, *p *< 0.05) and Posthypnotic amnesia (χ^2 ^= 6.09, OR = 2.28, 95% CI: 1.17 ~ 4.43, *p *< 0.05) significantly more often than did the healthy volunteers (see Table 3).

**Table 1:** Three-Way ANOVA results for the Parker Personality Measure (PERM) scale scores in personality disorder patients (n = 77) and healthy volunteers (n = 154).

Effect	df, de	F value	*p *value	MSE
**Group**	1, 227	76.34	0.00	6571.77
**Gender**	1, 227	1.62	0.20	139.62
**PERM**	10, 2270	223.88	0.00	5408.34
**Group, Gender interaction**	1, 227	5.99	0.02	516.06
**Group, PERM interaction**	10, 2270	5.17	0.00	124.81
**Gender, PERM interaction**	10, 2270	1.10	0.36	26.62
**Group, Gender, PERM interaction**	10, 2270	1.79	0.08	43.24

Note: df, degree of freedom; de, degree of error.

**Table 2:** Scale scores (Mean ± S.D.) of the Parker Personality Measure in personality disorder patients (n = 77) and healthy volunteers (n = 154).

	Personality Disorder	Healthy Control
**Paranoid**	27.96 ± 6.79	22.57 ± 5.57*
**Schizoid**	23.69 ± 5.44	19.42 ± 3.35*
**Schizotypal**	13.86 ± 4.52	9.95 ± 2.98*
**Antisocial**	24.75 ± 6.10	21.05 ± 4.46*
**Borderline**	26.45 ± 7.72	19.95 ± 5.27*
**Histrionic**	15.69 ± 4.22	13.62 ± 2.77*
**Narcissistic**	21.39 ± 5.24	18.37 ± 4.14*
**Avoidant**	30.45 ± 7.08	24.71 ± 5.39*
**Dependent**	25.31 ± 6.49	22.03 ± 4.56*
**Obsessive-Compulsive**	19.22 ± 4.76	17.21 ± 3.25*
**Passive-Aggressive**	24.38 ± 5.74	20.98 ± 4.50*

Note: * *p *< 0.01 vs. normal controls (post-hoc test after three-way ANOVA)

**Table 3:** Distribution of participants who passed or failed the hypnotic susceptibility tests in personality disorder patients (n = 77) and healthy volunteers (n = 154).

	Personality Disorder		Healthy control	
	Passed number, rate	Failed number, rate	*Passed* *number, rate*	*Failed* ***number, rate***
**Hand lowering**	55, 71.4%	22, 28.6%	*106, 68.8%*	*48, 31.2%*
**Moving hands apart**	52, 67.5%	25, 32.5%	*108, 70.1%*	*46, 29.9%*
**Mosquito hallucination**	44, 57.1%	33, 42.9%	*89, 57.8%*	*65, 42.2%*
**Taste hallucination**	55, 71.4%	22, 33.8%	*98, 63.6%*	*56, 36.4%*
**Arm rigidity**	52, 67.5%	25, 32.5%	*99, 64.3%*	*55, 35.7%*
**Dream**	38, 49.4%*	39, 50.6%	*55, 35.7%*	*99, 64.3%*
**Age regression**	65, 84.4%	12, 15.6%	*127, 82.5%*	*27, 17.5%*
**Arm immobilization**	42, 54.5%	35, 45.5%	*76, 49.4%*	*78, 50.6%*
**Anosmia to ammonia**	16, 20.8%	61, 79.2%	*39, 25.3%*	*115, 74.7%*
**Hallucinated voice**	7, 9.1%	70, 90.9%	*10, 6.5%*	*144, 93.5%*
**Negative visual hallucination**	23, 29.9%	54, 70.1%	*52, 33.8%*	*102, 66.2%*
**Posthypnotic amnesia**	22, 28.6%*	55, 71.4%	*23, 14.9%*	*131, 85.1%*

Note: *p < 0.05 vs. normal controls

When considering the prediction of PERM functioning styles by SHSSC items, no significant predictor was found in the healthy control group. In contrast, in the patient group, the accounted variance (adjusted R^2 ^values) by the significant correlations ranged from 0.04 to 0.09. SHSSC Posthypnotic amnesia significantly predicted the Schizoid (β = 0.25, adjusted R^2 ^= 0.05, *p *< 0.05), but negatively the Narcissistic style (β = -0.23, adjusted R^2 ^= 0.04, *p *< 0.05). Taste positively (β = 0.26, adjusted R^2 ^= 0.09, *p *< 0.05) and Anosmia to ammonia negatively (β = -0.23, adjusted R^2 ^= 0.09, *p *< 0.05) predicted the Borderline style.

## Discussion

Compared to the healthy volunteers, the patients scored higher on all PERM scales, and possessed higher passing rates on SHSSC Dream and Posthypnotic amnesia. In the patients, some SHSSC items were significantly correlated with the PERM scales. However, the adjusted R²s of these correlations were relatively low in both groups, suggesting that the correlation between hypnotic susceptibility and the functioning styles of personality disorder was weak. In some patients, the weak correlation might be due to their subconscious defense to hypnosis. As noticed earlier [18], when facing the examiner, participants would be sensitive to the potential threat to the ego and would mobilize their defense mechanisms. The pronounced correlation in patients might indicate that hypnotic susceptibility influences the expression of personality disorders.

The higher passing rates of SHSSC Dream and Posthypnotic amnesia, two cognition related items in our patients, were similar to a previous investigation which showed that people with mixed personality disorders had higher hypnotic susceptibility [7]. Specifically, the higher SHSSC Dream passing rate might help us to understand the prevalence of hallucination in personality disorders [19], and some scholars attributed Hypnotic amnesia to the conscious suppression of memory due to a defense mechanism [20], which might be particularly the case in patients with personality disorders who had traumatic experiences [21]. Consequently, the present results helped to understand the prevalence of hallucination and memory deficit in personality disorders [22].

Regarding hallucination in personality disorders, the borderline type would be a particular example. In our patients, PERM Borderline style was positively correlated with SHSSC Taste. This correlation might indicate that patients with borderline personality disorder features were prone to hallucination and to the autistic fantasy defense [23]. Indeed, the borderline personality disorder is commonly associated with hallucination [24], but this phenomenon was once overlooked [25].

According to one theoretic interpretation of hypnosis, Posthypnotic amnesia occurs because the forgotten materials are dissociated from awareness, and it denotes the most deteriorated stage of dissociation [20]. When referring to the Schizoid personality disorder, patients are likely to pay little attention to how they behave, or how their behavior may or may not impress the experimenter [26]. Consequently, they might easily forget the experience obtained during the experiment. Moreover, with reversibility as an essential mark, Posthypnotic amnesia is somewhat like the temporarily retrograde amnesia. Studies have consistently reported the retrograde amnesias in patients with impairment in the frontal lobe [27]. Meanwhile, the schizoid personality disorder patients also displayed neuropsychological malfunctions in relation to the frontal lobe [28]. This might be a possible mechanism behind the correlation between the PERM Schizoid and the SHSSC Posthypnotic amnesia in our patients.

On the other hand, patients with narcissistic personality disorder requiring excessive admiration from others, are likely to participate in tasks which merit special talents, to display a strong intense reaction to perceived threats to self-esteem, are sensitive to criticism, and are known to be active and flamboyant [29]. Therefore, they might be particularly interested in what the experimenter had ordered, and in how their actions may impress the experimenter. This could be particularly likely when they are asked to recall the details of the experiment. Thereupon, lower SHSSC Posthypnotic amnesia would be correlated with the higher PERM Narcissistic style in patients with personality disorders.

Nonetheless, several limitations of the present study design are noted. We used the Chinese version of SHSSC which has not yet been validated. We did not divide our personality disorder patients into individual types, and the age spans of our participants were narrow. In addition, Axis I disorders such as anxiety, depression and sleep disorders were not included in the present study. Furthermore, we found correlations (predictions) which were in one direction only, and these correlations in both our groups were low. Nevertheless, our findings could help to explain the psychotic features in personality disorders such as hallucination and memory distortion, and support the use of Psychoanalytic therapy in this pathology, regardless of its intractability [30]. It has also been shown that the hypnotic technique in particular could reduce a half treatment course for personality disorders [31]. Although the defense mechanism of a participant is constant [18], our findings imply at least that a memory retrieval may help to normalize the functioning style of Schizoid personality disorder, while an external negative experience (e.g., ammonia) exposure may help to normalize the style of the Borderline disorder.

## Conclusion

Our results indicate that in personality disorder patients, the hypnotic susceptibility influences their personological functioning styles in general. Future studies might be designed to see the detailed correlation patterns in different subtypes of personality disorder.

## List of abbreviations

SHSSC: the Stanford Hypnotic Susceptibility Scale, Form C; PERM: the Parker Personality Measure; PTSD: posttraumatic stress disorder; DID: dissociative identity disorder.

## Competing interests

The authors declare that they have no competing interests.

## Authors' contributions

FW, WC, JH, and PX conducted the hypnotic susceptibility tests on participants, WH, HC, JZ, and WY collected the inventory data in students, WW and LC participated in the design and coordination of the study, and FW, WC, HW and WW drafted the manuscript. FW and WC contributed equally to the paper. All authors read and approved the final manuscript.

## Pre-publication history

The pre-publication history for this paper can be accessed here:

http://www.biomedcentral.com/1471-244X/11/121/prepub
